# The Role of Mitonuclear Incompatibility in Bipolar Disorder Susceptibility and Resilience Against Environmental Stressors

**DOI:** 10.3389/fgene.2021.636294

**Published:** 2021-03-16

**Authors:** Suzanne Gonzalez

**Affiliations:** Department of Psychiatry and Behavioral Health, Department of Pharmacology, Penn State College of Medicine, Hershey, PA, United States

**Keywords:** bipolar disorder, genetics, mitonuclear coevolution, mitonuclear coadaptation, mitonuclear incompatibility, mitonuclear interaction, epistasis

## Abstract

It has been postulated that mitochondrial dysfunction has a significant role in the underlying pathophysiology of bipolar disorder (BD). Mitochondrial functioning plays an important role in regulating synaptic transmission, brain function, and cognition. Neuronal activity is energy dependent and neurons are particularly sensitive to changes in bioenergetic fluctuations, suggesting that mitochondria regulate fundamental aspects of brain function. Vigorous evidence supports the role of mitochondrial dysfunction in the etiology of BD, including dysregulated oxidative phosphorylation, general decrease of energy, altered brain bioenergetics, co-morbidity with mitochondrial disorders, and association with genetic variants in mitochondrial DNA (mtDNA) or nuclear-encoded mitochondrial genes. Despite these advances, the underlying etiology of mitochondrial dysfunction in BD is unclear. A plausible evolutionary explanation is that mitochondrial-nuclear (mitonuclear) incompatibility leads to a desynchronization of machinery required for efficient electron transport and cellular energy production. Approximately 1,200 genes, encoded from both nuclear and mitochondrial genomes, are essential for mitochondrial function. Studies suggest that mitochondrial and nuclear genomes co-evolve, and the coordinated expression of these interacting gene products are essential for optimal organism function. Incompatibilities between mtDNA and nuclear-encoded mitochondrial genes results in inefficiency in electron flow down the respiratory chain, differential oxidative phosphorylation efficiency, increased release of free radicals, altered intracellular Ca^2+^ signaling, and reduction of catalytic sites and ATP production. This review explores the role of mitonuclear incompatibility in BD susceptibility and resilience against environmental stressors.

## Introduction

Mitochondria are multifunctional organelles found in large numbers in almost all human cells. They are highly effective in producing more than 90% of the cellular energy needed by the body in the form of ATP (Wallace, 2005). Efficient mitochondrial functioning depends on synchronized gene expression and protein interactions transcribed from both the mitochondrial (mtDNA) and nuclear (nDNA) genomes. This synchronization between genomes is essential for energy production needed to sustain life and organ function in addition to regulating a wide range of cellular pathways including homeostatic regulation of ionic gradients, cell signaling, and cellular stress response and survival (Mattson et al., 2008). The mitochondria genome encodes 13 proteins of the electron transport chain (ETC), 22 transfer RNAs, and 2 ribosomal RNAs (Anderson et al., 1981). Other mitochondrial proteins are encoded in the two copies nDNA. Individual mitochondrion typically include 2–10 mtDNA copies, while the number of mitochondria per cell varies up to 1,000 (Robin and Wong, 1988) depending on energy demands, oxidative stress and pathological conditions (Clay Montier et al., 2009).

Mitochondrial function is particularly important in neurons due to the high bioenergetic demands of neuronal activities (Chan, 2006; Mattson et al., 2008). Mitochondrial functioning plays an important role in regulating synaptic transmission, brain function, and cognition (Picard and McEwen, 2014). Neuronal activity is energy-dependent, and neurons are particularly sensitive to changes in bioenergetic fluctuations, suggesting that mitochondria regulate fundamental aspects of brain function. Mitochondrial deficits including energy production, oxidative stress, oxidation-reduction (redox) imbalance, and Ca^2+^ metabolism, have been implicated in the etiology of psychiatric diseases (Manji et al., 2012; Park and Park, 2012; Srivastava et al., 2018). It has long been postulated that disruption of mitochondrial function contributes to the etiology of bipolar disorder (BD). Kato and Takahashi (1996) and Kato et al. (1997a, b) demonstrated leukocytes and autopsied brains from BD individuals exhibited increased levels of the 4,977-bp mtDNA deletion, as well as abnormal brain energy metabolism in BD including decreased intracellular pH (Kato et al., 1993, 1998; Hamakawa et al., 2004), decreased phosphocreatine (Kato et al., 1992, 1994, 1995), and enhanced response of phosphocreatine in lithium-resistant BD (Murashita et al., 2000). These findings led to the mitochondrial dysfunction hypothesis of BD in 2000, theorizing that “mtDNA mutations as well as common variations may confer a risk of BD by affecting intracellular calcium signaling systems” (Kato and Kato, 2000). Since then, mitochondrial abnormalities associated with bipolar disorders have been extensively reviewed at the structural, molecular and functional levels (Kato and Kato, 2000; Kato, 2005, 2006, 2008, 2017; Stork and Renshaw, 2005; Quiroz et al., 2008; Shao et al., 2008; Clay et al., 2011; Konradi et al., 2012; Kim et al., 2015; Duong et al., 2016; Machado et al., 2016; Scaini et al., 2016; Cikankova et al., 2017; Morris et al., 2017; Andreazza et al., 2018; Holper et al., 2018; Srivastava et al., 2018), as well potential therapeutic strategies (Kato, 2007; Wang, 2007; Quiroz et al., 2008; Nierenberg et al., 2013; de Sousa et al., 2014a; Callaly et al., 2015; Cikankova et al., 2017; Pereira et al., 2018).

While there is mounting evidence supporting the mitochondrial dysfunction hypothesis of BD, recent BD GWAS have failed to show significant association with mtDNA and/or nDNA mitochondria related genes (Prata et al., 2019; Stahl et al., 2019). The closest BD risk allele to a mitochondrial core gene is rs74446114, located 63.5 kb downstream of *MRPL33*, which encodes Mitochondrial Ribosomal Protein L33 (Stahl et al., 2019). A greater understanding of the core evolutionary processes driving mitonuclear and environmental interactions will provide key insights into the complex etiology underlying mitochondrial dysfunction in BD.

## Co-Evolution and Co-Adaptation of Mitonuclear Genomes

Mitochondria are double-membrane structures with their own maternally inherited, circular genome (16,568 bp) consisting of transcription, translation, and protein assembly systems conserved from its endosymbiotic origin (Anderson et al., 1981; Gray et al., 1999). In the course of evolution, mitochondria became dependent on the nuclear genome for nuclear-encoded factors necessary for its integrity, replication and expression (Gray et al., 1999). While 13 mitochondrial proteins are encoded by mtDNA, approximately 1,158 mitochondrial proteins are encoded by nDNA and imported into the mitochondrion (Poyton and McEwen, 1996; Calvo et al., 2016). Studies suggest that mitochondrial and nuclear genomes co-evolve to ensure the co-adaptation and coordinated expression of interacting proteins vital for mitochondrial biosynthesis and organism survival (Cosmides and Tooby, 1981; Bar-Yaacov et al., 2012).

Mitonuclear allelic interactions are essential to maintain evolutionary co-adaptation. Nuclear genes coding for mitochondrial-related proteins that co-function with mtDNA gene products, must co-evolve with arising deleterious mtDNA variants (Hill, 2016). If a mutation arises in mtDNA, the nuclear genome needs to adapt (i.e., nuclear mutation needs to arise to off-set the mtDNA mutation) in order to restore the synergistic interactions between these two genomes. MtDNA are more susceptible to SNPs, indels, and structural changes compared to their nDNA counterparts, as it has a higher mutation rate, lacks recombination, and has an inefficient DNA repair mechanism. As deleterious mutations reach a frequency threshold, the host fitness becomes compromised. As these deleterious mtDNA mutations spread through a population, other mutations accumulate to restore the host fitness by either directly compensating the effect of the mtDNA mutation in the nDNA or by inducing weak compensatory mutations in both genomes to try to restore fitness through selection (see Chou and Leu, 2015 for review). This mitonuclear co-evolution results in differential accumulation of population-specific mitonuclear mutations over time. While some studies suggested that most mtDNA variants in populations are selectively neutral to prevent removal by selective pressure (Gregorius and Ross, 1984; Wallace et al., 1999), the question remains whether this selective neutrality is dependent on the ancestral nuclear background.

**FIGURE 1:**
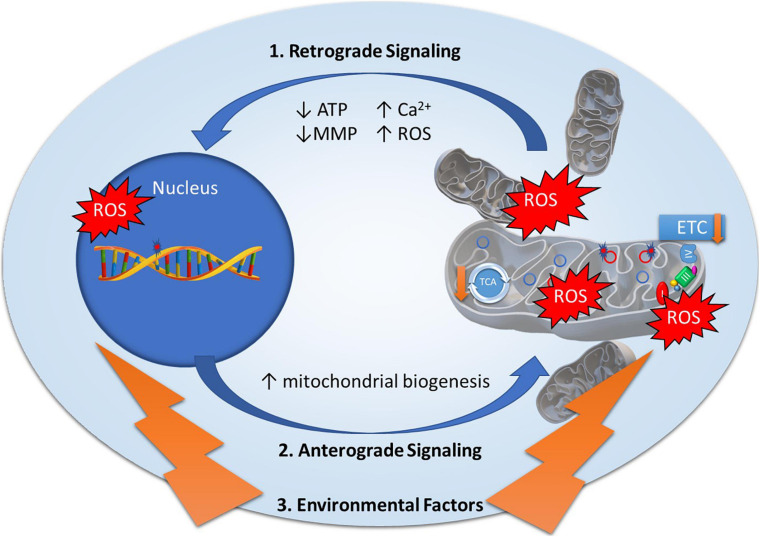
Mitonuclear communication in bipolar disorder. Mitochondrial dysfunction in bipolar disorder has been attributed to genetic variants in mitochondrial DNA and nuclear-encoded mitochondrial genes as well as decreased electron transport chain (ETC) and tricarboxylic acid (TCA) cycle activity. Mitochondrial function is under the regulation of retrograde and anterograde signals and adaptive responses to environmental factors. ***1. Retrograde signaling*** (mitochondria to nucleus) activated by mitochondrial stress signals including Adenosine Triphosphate (ATP), Reactive Oxygen Species (ROS), Mitochondrial Membrane Potential (MMP), and calcium levels (Ca^2+^) to regulate nuclear gene expression to restore mitochondrial homeostasis. ***2. Anterograde signaling*** (nucleus to mitochondria) are nucleus-controlled regulation of gene expression to modulate mitochondrial activity, mitochondrial biogenesis, and mitochondrial fusion/fission. ***3. Environmental factors*** such as diet, exercise, and temperature elicit adaptive responses to modulate mitochondrial stress signals and expression of nuclear encoded mitochondrial related genes.

Mitonuclear genomes are under the regulation of retrograde and anterograde “cross-talk” signals to modulate mitochondrial function (Figure 1), most often originating from the mitochondria to induce specific nuclear responses to regulate mitochondrial structure and function (Quiros et al., 2016). Mitochondria generate retrograde response signals to the nucleus to modulate nDNA gene expression in response to mtDNA mutations and to maintain cellular function, metabolism, and adaptation to environmental stressors such as diet, exercise, and temperature (Quiros et al., 2016). Alternatively, anterograde regulation sends signals from the nucleus to the mitochondria in order to regulate mitochondrial activity and promote mitochondrial biogenesis through cell growth and survival. Anterograde signaling can modulate bioenergetics based on cellular demands via mitochondrial fusion/fission to regulate mitochondrial copy number, morphology, mitophagy, and movement (Chen and Chan, 2009; Chan, 2012; Quiros et al., 2016). Mutations in any of these essential components may disrupt cross-talk between mitonuclear genomes, affecting mitochondrial stability and function, and ultimately contributing to diseases (Poyton and McEwen, 1996).

It has been suggested that population admixture may introduce mitonuclear discordance, in which mitochondrial and nuclear genomes originate from differentiated ancestries. Chou and Leu (2015) argued that if co-evolution in mtDNA and nDNA commonly occur in diverse populations, the balance between deleterious mtDNA mutations and compensatory nDNA variants may be dependent on their nuclear ancestral background, which may lead to genetic incompatibility between populations (Chou and Leu, 2015; Rand and Mossman, 2020). Individuals who have ancestors from different continental populations may have certain mtDNA genetic variants from one continental population, but might not have the corresponding adaptive mutation in the nuclear genome from the same continental population. Zaidi and Makova (2019) recently reported a significant increase in the local genetic ancestry at mitochondrial related genes in nDNA toward the corresponding mtDNA ancestry in African American and Native American admixed populations, providing empirical evidence that selective pressure helps restore mitonuclear compatibility in human admixed populations.

The evidence for differential lifetime prevalence of BD based on race/ethnicity is conflicting (Grant et al., 2005; Substance Abuse and Mental Health Services Administration, 2020) and specific prevalence of BD in admixed individuals are lacking. However, results from the *2019 National Survey on Drug Use and Health* reports that the prevalence of any mental illness (AMI) and severe mental illness (SMI) is highest among adults reporting two or more races (AMI = 31.7% compared to 22.2% White adults; SMI = 9.3% compared to 5.7% White adults) (Substance Abuse and Mental Health Services Administration, 2020), suggesting admixed individuals are at higher risk for SMI, including BD. SMI is also higher in American Indian/Alaska Natives (6.7%) compared to White adults (Substance Abuse and Mental Health Services Administration, 2020). Lifetime mood disorders are significantly more prevalent among the American Indian/Alaska Natives population compared to Whites, with American Indian/Alaska Natives men showing greater trend toward BDI and women toward BDII (Brave Heart et al., 2016). American Indian/Alaska Natives admixed with two or more races reported a higher lifetime prevalence of diagnosed depressive disorder, more days of poor mental health, and more frequent reporting of mental distress compared to both American Indian/Alaska Natives-single race and White-single race groups. These results remained significant after adjustment for sociodemographic covariates (Asdigian et al., 2018), suggesting that increased prevalence mood disorders and related clinical characteristics reported in American Indian/Alaska Natives may be caused in part by increased population admixture.

While current evidence of mitonuclear discordance stems from crosses of various model organisms (Ballard, 2005; Kurbalija Novicic et al., 2015; Mossman et al., 2016b; Loewen and Ganetzky, 2018), human cytoplasmic hybrids (cybrids) (Esteves et al., 2008; Lin et al., 2012; Silva et al., 2013; Kenney et al., 2014a; Nashine et al., 2016; Shrivastava and Subbiah, 2016; Zhou et al., 2017) and conplastic animals (Yu et al., 2009; Weiss et al., 2012; Kumarasamy et al., 2013; Houstek et al., 2014; Latorre-Pellicer et al., 2016) studies are increasingly being used to demonstrate the mitonuclear effects of mtDNA variants in different nuclear ancestral backgrounds. Cybrids and conplastic animals are constructed using identical nuclei and external mitochondria with different mtDNA haplogroups in order to investigate resulting bioenergetic and physiological effects. These models also allow for investigations into environment-specific modulation of intraindividual mitonuclear interactions (Dowling et al., 2007; Soh et al., 2007; Zhu et al., 2014; Mossman et al., 2017; Rand et al., 2018; Dong et al., 2019).

## Oxidative Phosphorylation Dysregulation in Bipolar Disorder

Mitochondria generate cellular energy through the OXPHOS system via the ETC, consisting of five multiprotein complexes (complex I–V) within and across the inner mitochondrial membrane. Thirteen ETC proteins are encoded by mtDNA genes, while > 1,000 proteins that make up the mitochondria are encoded by genes in the nuclear genome (Mattson et al., 2008; Calvo et al., 2016). OXPHOS involves respiration, an oxidative exergonic pathway, which leads to the phosphorylation of ADP into ATP. OXPHOS dysregulation has been previously reported in BD (Konradi et al., 2004; Iwamoto et al., 2005; Sun et al., 2006; Beech et al., 2010). Konradi et al. (2004) reported a significant decrease in mRNA expression levels of mitochondrial genes in the hippocampus of BD subjects, including genes regulating OXPHOS and proteasomal degradation. Iwamoto et al. (2005) found that 15.1% of 676 mitochondria-related genes were differentially expressed in BD. However, they concluded the reported global downregulation of mitochondrial genes was possibly a consequence of pharmaceutical effects, as BD medication-naive patients exhibited an up-regulation of a subset of 27 mitochondrial-related genes, including *COX15*, *UQCRC2*, *ETFDH*, and *NDUFS1* involved in mitochondrial respiratory chain (Iwamoto et al., 2005). Using a modified Gene Set Enrichment Analysis (GSEA) computational screen to estimate collaborative candidate pathways co-expressed with OXPHOS dysregulation related to BD pathogenesis, Sawai et al. (2015) suggested that aberrant mitochondrial OXPHOS may induce dysregulation in the ubiquitin-proteasome pathway. Another study reported susceptibility to bioenergetic alterations within distinct brain regions in BD. They reported significant reductions in mitochondrial function in the PFC of patients with BD and increase in function in the hypothalamus and cerebellum, suggesting that mitochondrial dysfunction in brain specific regions may lead to increased oxidative stress, thus triggering compensatory mechanisms to offset oxidative damage of mtDNA (Bodenstein et al., 2019).

While several studies have reported associations of BD with various mtDNA variants and mitochondrial haplogroups (Kato et al., 2001; Munakata et al., 2004; Kazuno et al., 2009; Rollins et al., 2009; Frye et al., 2017), no mtDNA variants have reached GWAS thresholds of significance. In addition, mitonuclear co-adaptation could result in spurious associations of disease with mtDNA variants due to population stratification (Hagen et al., 2018). Several researchers have postulated that investigations focused on mitonuclear epistatic interactions may reveal additional genes associated with BD risk. Ryu et al. (2018) reported mitonuclear epistatic allelic interactions with nominal evidence for BD risk and/or early-onset BD based on European American GWAS data from the GAIN BD study. They reported nominal evidence of epistatic associations involving mitochondrial encoded ETC complex I genes mt-*CytB* and mt-*ND4* with BD risk and risk of early onset BD. Mitonuclear epistatic allelic interactions associated with BD risk were reported for mt-*CytB* and *MGAM*, mt-*CytB* and *AK5*, and mt-*ND4* and *MGAM*. For early onset BD risk among BD patients, the top association signals were detected in mt-*CytB* and *CTNNA2*, mt-*CytB* and *IL34, and mt-ND4* and *IL34* interactions (Ryu et al., 2018). Schulmann et al. (2019) reported on the association between the risk of SZ and mitonuclear allelic interactions in an Irish cohort. They reported 34 mitonuclear epistatic allelic interactions with significant joint effects between 10 mitochondrial and 21 nuclear genes, where six of the mitochondrial genes encoded ETC complex I components (Schulmann et al., 2019).

Several cybrid studies have utilized mtDNA-naive ρ^0^ human cells fused with platelets resulting in cell lines with identical nuclei and different mtDNA haplogroups in order to evaluate mtDNA variant and haplotype effects on mitochondrial energy production (Kenney et al., 2014a, b; Zhou et al., 2017). Zhou et al. (2017) reported several mtDNA SNPs associated with differential endogenous mitochondrial respiratory activity and uncoupled mitochondrial respiration. Different East Asian haplogroups exhibited differential effects on mtDNA copy number (mtDNAcn), transcription efficiency, complex III expression, and coupled and uncoupled mitochondrial respiration (Zhou et al., 2017). Kenney et al. compared OXPHOS related gene expression of mtDNA-encoded respiratory complex genes between West Asian and European mitotype cybrids (J and H haplotypes, respectively). West Asian mitotype cybrids exhibited lower OXPHOS utilization compared to European mitotype cybrids. They also compared cybrids with African, European, and West Asian mitotypes haplogroups (L, H, and J haplotypes, respectively). Haplogroups exhibited unique bioenergetic profiles with differential effects on mtDNAcn, OXPHOS gene expression, ATP turnover, and spare respiratory capacity. African mitotype cybrids displayed more efficient OXPHOS system, as evidenced by higher expression levels of mtDNA-encoded respiratory complex genes, decreased ATP turnover rates and lower reactive oxygen species (ROS) production (Kenney et al., 2014b). Cybrids with European mitotype had elevated ATP production and decreased lactate levels compared to West Asian mitotypes cybrids (Kenney et al., 2013). West Asian mitotypes cybrids exhibited altered bioenergetic profiles compared with European mitotype cybrids, including significantly decreased expression in mtDNA encoded respiratory genes encoded by mtDNA. In addition, West Asian and European mitotype cybrids had significantly altered expression of eight nuclear genes involved in inflammatory response and apoptosis (Kenney et al., 2013, 2014a). While these studies proposed that effects are dependent on mitochondrial haplotypes, the underlying ancestries of the ρ^0^ cells used in these studies must also be taken into consideration. For example, Zhou et al. (2017) utilized 143B ρ^0^ cells which are derived from an individual with an admixed nuclear genetic ancestral background consisting of South European (59%), South Asian (37%), and South East Asian (4%) genomic ancestries (Dutil et al., 2019) and compared the effects of differing East Asian mtDNA genetic backgrounds. Differences in OXPHOS and mitochondrial bioenergetics may reflect disruptions in co-adaptation of population specific mitonuclear interactions rather than effects of mitochondrial variants or haplotypes alone.

While the use of human cybrids are commonly used to study mitochondrial dysfunction in neurodegenerative disorders such as Alzheimer’s (Onyango et al., 2006; Yu et al., 2016, 2017; Weidling et al., 2020), and Parkinson’s disease (Keeney et al., 2009; Cronin-Furman et al., 2013; Reeve et al., 2015; Pignataro et al., 2017), there is a paucity of cybrid research in BD and other psychiatric disorders. Munakata et al. (2004) reported that the m.3644T > C variant in the mt-*ND1* gene was associated with BD and 3644C cybrids exhibited mitochondrial dysfunction including decreases in mitochondrial membrane potential (MMP) and complex I activity compared to 3644T cybrids. Verma et al. (2016) determined that cybrids developed by fusing platelets from ADHD patients with SH-SY5Y ρ^0^-cells resulted in altered mitochondrial bioenergetics including significant decreases in mitochondrial respiration, ETC complex V activity, MMP, and increases in oxidative stress. A recent study reported potential therapeutic benefits of mitochondrial transplantation in SZ. Robicsek et al. (2018) demonstrated that the transfer of healthy mitochondria into SZ-iPSC differentiated neurons and rat SZ-models increased mtDNAcn and improved cellular respiration, MMP, mitochondrial distribution, and neuronal differentiation.

Compelling evidence in animal models suggests that population admixture with differing mtDNA can intensify oxidative stress and DNA damage, affect fitness, and bioenergetic activity due to mitonuclear discordance, and these effects can be modulated by environmental factors including temperature, diet, and exercise. In copepod (*Tigriopus californicus)* populations, Barreto and Burton (2013) studied 28 hybrid strains derived from 12 parental lines. Hybrids displayed a significant decrease in fitness and enhanced oxidative stress compared to the parental strains. In addition, admixed populations that showed evidence of decreased fecundity exhibited enhanced oxidative stress compared to those with no discernable change in fitness. Hybrid strains with high levels of mitonuclear discordance exhibited significant increases oxidative DNA damage, while hybrid lines with low mitonuclear discordance showed no significant difference from parental lines (Barreto and Burton, 2013).

Baris et al. (2016) reported persuasive evidence that natural selection to variable thermal environments maintains mitonuclear interactions regulating OXPHOS metabolism in admixed populations of mummichog fish (*Fundulus heteroclitus*) with northern or southern mitochondrial haplotypes. Effects of acute temperature change were dependent on northern and southern mitochondrial haplotypes. While fish with the southern mt-haplotype had acute effects in OXPHOS metabolism at low and high acclimation temperatures, little to no acute temperature effect was reported with fish with the northern mt-haplotype. Significant differential responses to acute temperature change were dependent on mitochondrial haplotypes at high acclimation temperatures for ADP stimulated respiration, mitochondrial uncoupling, and complex I and II respiration (Baris et al., 2016). Mummichog fish with a high level of mitonuclear discordance with northern mt-haplotype exhibited enhanced OXPHOS efficiency while those with a southern mt-haplotype showed decreased OXPHOS efficiency (Baris et al., 2017).

Environmental factors have also been shown to modulate mitonuclear responses in *Drosophila.* Hoekstra et al. (2013) crossed *D. simulans* with *D. melanogaster* to create four distinct mitonuclear genotype strains by combining two mtDNA alleles with two nuclear alleles previously shown to exhibit epistatic mitonuclear interactions (Montooth et al., 2010). One of the mitonuclear genotype strains resulted in delayed development, decreased fitness, height reduction, and inefficient bioenergetics that were directly proportional to temperature (Hoekstra et al., 2013). Montooth et al. (2019) also demonstrated that mitonuclear incompatibility in *Drosophila* adversely affects fertility in males developed at warmer temperatures and these deleterious effects could be partly reversed by diet. Dowling et al. (2007) reported significant mitonuclear interactions contributed to adult female fitness in a *D. melanogaster* population, in which main effects could not be attributed to mitotype or nuclear background alone. They also reported that minor variations in environment including temperature, humidity, and diet contributed to mitonuclear effects on relative fitness (Dowling et al., 2007). Soh et al. (2007) studied the effects of dietary intake on normal and extended longevity strains as well as introgressed mitonuclear hybrid strains in *Drosophila*. The extended longevity strains exhibited lower levels of ROS, enhanced mitochondrial bioenergetic efficiency, heightened antioxidant response, and reduced oxidative damage (Arking et al., 2002). Nutrition levels resulted in differential longevity dependent on mitonuclear combinations. Hybrids with the more efficient “extended longevity” mitochondria exhibited enhanced longevity in males but decreased longevity females under non-starvation nutritional diets (Soh et al., 2007). A study utilizing admixed *D. simulans* strains with differing mitotypes from mitonuclear compatible control strains also concluded that compensation for mitonuclear discordance may be temperature-dependent (Pichaud et al., 2013).

Investigations involving introgressive hybridized seed beetles (*Callosobruchus maculatus*), also investigated mitonuclear and environmental effects on fitness. Three mitochondrial haplotypes were backcrossed with nuclear genetic backgrounds from Brazil, California, and Yemen. The three mitotypes displayed significant differences in relative fitness. Strains with concordant mitonuclear genomes exhibited increased relative fitness compared to strains with differing mitonuclear genomes. At colder temperatures, Brazilian and Yemen mitotypes introgressed with California nuclear genomes showed opposite effects in fitness, again suggesting that temperature contributes to mitonuclear effects on relative fitness (Immonen et al., 2020).

Kumarasamy et al. (2013) constructed conplastic rat strains using two common rat models for cardiovascular disease, in which the mtDNA of the Dahl salt-sensitive (S) rat was exchanged with the mtDNA of the spontaneously hypertensive rat (SHR) and vice versa. The S.SHR^mt^ and SHR.S^mt^ conplastic strains displayed significant increases in mtDNAcn, decreases in mitochondrial ROS levels, and increases in aerobic fitness and survival compared to the S rat. The S.SHR^mt^ conplastic strain also showed significant increases in mtDNAcn compared to the S rat (Kumarasamy et al., 2013). In toto, these results indicate that cellular bioenergetics, mtDNAcn, transcription efficiency, fitness, and survival are greatly affected by mitonuclear interactions modulated by environmental factors.

## Altered Mitochondrial Calcium Regulation in Bipolar Disorder

Mitochondria play a critical role in intracellular Ca^2+^ homeostasis and Ca^2+^-mediated signaling processes (Mattson et al., 2008). Mitochondrial dysregulation is more pronounced in neurons, as the brain is the largest source of energy consumption due to the high bioenergetic demands of neuronal functions, and can lead to an imbalance in calcium homeostasis (Chan, 2006; Mattson et al., 2008). Mitochondria regulate rapid changes in intracellular Ca^2+^ dynamics. Increases in intracellular Ca^2+^ and uptake into the mitochondria matrix results in altered mitochondrial membrane permeability, ETC efficiency, and Ca^2+^-mediated signaling processes modulating gene expression, leading to dysregulation of several neuronal systems including neurotransmitter release, regulation of neuronal action potentials, dendritic developmental and remodeling, synaptic plasticity, regulation of gene expression, and apoptosis (Greer and Greenberg, 2008; Feissner et al., 2009; Peng and Jou, 2010; Trevelyan et al., 2010; Srivastava et al., 2018), and can alter neuronal function through the production ROS (Sena and Chandel, 2012).

Altered mitochondrial calcium regulation may contribute to the pathophysiology of neuropsychiatric disorders including BD (Kato et al., 2003; Kazuno et al., 2006; Kubota et al., 2006; Kato, 2007, 2008). Mertens et al. (2015) reported direct correlations between mitochondria defects, altered Ca^2+^ signaling, and hyperexcitability of neurons in BD. Hippocampal neurons differentiated from iPSCs from BD lithium responders and non-responders exhibited enhanced mitochondrial function as evidence by higher MMP and upregulated mitochondrial gene expression compared to control neurons. Ca^2+^ signaling pathways were altered in BD neurons compared to controls. Based on patch-clamp recording and somatic Ca^2+^ imaging in BD neurons, increased mitochondrial activity resulted in neuronal hyperexcitability, which was reduced by lithium in neurons from BD lithium responders, but not in BD non-responders (Mertens et al., 2015).

Kazuno et al. (2006) examined the phenotypic effect of mtDNA using calcium indicator cybrids 143B.TK^–^ρ^0^206 that stably expresses two ratiometric fluorescent proteins. Using these cybrids, they identified two mtDNA variants which altered mitochondrial pH and calcium concentration. In fluorescent images of cybrids, mitochondria exhibited increased calcium concentrations and pH in the mitochondrial matrix compared to cytosolic levels, suggesting m.10398A in the mt-*ND3* gene of complex I and m.8701A in the ATPase6 gene of complex V alter mitochondrial pH and intracellular calcium homeostasis. Cytosolic calcium response to histamine stimulation also differed between cybrid cells carrying the m.10398A and m.8701A variants (Kazuno et al., 2006). The m.10398A mutation has been reported to be associated with BD (McMahon et al., 2000; Kato, 2001; Kato et al., 2001; Washizuka et al., 2003), as well as valproate (Kazuno et al., 2008) and lithium response (Washizuka et al., 2003), which suggests that these variants may contribute to the etiology of BD.

## Mitochondrial Dna Copy Number in Bipolar Disorder

Alterations in mtDNAcn signify changes in mitochondrial bioenergetics, energy demands, and compensatory effects (Picard et al., 2018). Several studies have found altered mtDNAcn in BD patients compared to controls. Significantly lower mtDNAcn have been observed in BD leukocytes (Chang et al., 2014; Yamaki et al., 2018; Wang et al., 2020), postmortem hippocampus (Fries et al., 2019), and left frontopolar cortex (Tsujii et al., 2019). Chung et al. (2020) reported lower mtDNAcn in BDI patients compared to higher mtDNAcn in BDII patients compared to controls. However, Yamaki et al. (2018) reported that both BDI and BDII patients had significantly decreased mtDNAcn versus controls. In contrast, de Sousa et al. failed to find significant differences in mtDNAcn in BD subjects compared to controls or before and after lithium treatment. They did, however, report a trend for decreased mtDNAcn in BDI compared to controls and BDII (de Sousa et al., 2014b). Wang et al. (2018) reported a significant reduction in leukocyte relative mtDNAcn in BD patients with manic and depressive symptoms compared to controls. There were no significant changes in mtDNAcn between euthymic BD patients and controls. In addition, they found that mtDNAcn was negatively correlated with total number of manic episodes (Wang et al., 2018). While higher levels of mtDNAcn in BD have also been reported (Fries et al., 2017), a meta-analysis for previous BD-mtDNAcn studies (Chang et al., 2014; de Sousa et al., 2014b; Fries et al., 2017; Wang et al., 2018; Yamaki et al., 2018) did not find any significant association with mtDNAcn, although mtDNAcn was significantly decreased in BD patients in an Asian-specific meta-analysis (Yamaki et al., 2018). A post-mortem study of suicide completers reported higher mtDNAcn in peripheral blood and decreased mtDNAcn in post-mortem PFC samples from suicide victims versus controls (Otsuka et al., 2017).

Zaidi and Makova reported that mtDNAcn was negatively correlated with mitonuclear discordance across mtDNA haplogroups of different geographic origins, consistent with mitonuclear incompatibility in admixed individuals. The authors also reported enrichment of ancestry at nuclear-encoded mitochondrial genes in African Americans and Puerto Ricans toward the corresponding ancestry of mtDNA haplogroups, suggesting a compensatory method of selection in restoring mitonuclear compatibility (Zaidi and Makova, 2019).

Human cybrid lines also show variation in mtDNAcn. Cybrids containing mtDNA variants m.249del, m.13708A, m.13928C, and m.16304C resulted in a decrease in mtDNAcn, while cybrids encompassing variants m.489C, m.8701G, m.10398G, and m.10400T resulted in increased mtDNAcn. The majority of these SNPs are diagnostic of a specific mtDNA haplogroup in East Asia mtDNAcn (Zhou et al., 2017). Variants m.13708A and m.13928C displayed decreased mtDNAcn compared to other SNPs, and have been previously associated with increased risk of disease (Yu et al., 2008; Korkiamaki et al., 2013), while m.489C, m.10398G, and m.10400T exhibit increased mtDNAcn and have been reported to have protective effects against disease (Darvishi et al., 2007).

BD environmental stressors have also been associated with alterations mtDNAcn. Lower mtDNAcn have been reported in response to maternal psychosocial stress, pollution, poor dietary (Ma et al., 2020) and inactive lifestyles, and tobacco use (Brunst et al., 2017; Wong et al., 2017; Kaali et al., 2018; Revesz et al., 2018; Breton et al., 2019; Wu et al., 2019; Duan et al., 2020; Hu et al., 2020; Vyas et al., 2020). In contrast, prior evidence indicates an increase in mtDNAcn in relation to history of early life stress and childhood trauma (Cai et al., 2015; Tyrka et al., 2016; Ridout et al., 2019, 2020). Discrepancies in the literature pertaining to mtDNAcn alterations in both BD and environmental modulators, specifically stressful life events, may be explained in part by tissue-specific alterations. Baek et al. (2019) investigated the effects of chronic immobilization stress in C57BL/6 male mice on mtDNAcn in 12 tissues. While they reported that chronic stress resulted in an increase in mtDNAcn in leukocytes, mtDNAcn was significantly decreased in prefrontal cortex, suggesting that mtDNAcn variability is tissue-specific (Baek et al., 2019).

## Discussion

While mtDNA variants can contribute to disease in humans (Taylor and Turnbull, 2005; Tuppen et al., 2010) and mtDNA haplogroups have been reported to associate with various disorders including psychiatric (Magri et al., 2007; Kazuno et al., 2009; Rollins et al., 2009), cardiovascular (Benn et al., 2008; Veronese et al., 2019), and metabolic diseases (Chinnery et al., 2007; Tavira et al., 2014; Mitchell et al., 2017; Zhao et al., 2019), these studies fail to encompass the complexity surrounding mitonuclear epistasis and environment interactions (Mossman et al., 2016a). Mitonuclear genetic variation embodies extraordinarily complex epistatic, pleiotropic and GxE interactions that function in non-additive ways to modify organismal fitness and survival. It has been argued that models optimized to study mitonuclear and environmental interactions are needed to better understand the underlying genetic architecture of complex phenotypes and to evaluate the nature of the polygenic models governed by mitonuclear cross-talk (Friedman and Nunnari, 2014; Chou and Leu, 2015; Quiros et al., 2016; Rand and Mossman, 2020).

This review postulates that disruptions in the co-evolution of the mtDNA and nuclear genomes, in addition to environmental factors, leads to mitochondrial impairment and increased risk of BD. Identification of the evolutionary mechanisms and genetic etiology underlying mitochondrial dysfunction is important in order to better understand the pathophysiology of the disease, to identify novel biomarkers of BD, to develop medications for treatment of BD, and to improve diagnostic predictive testing in diverse populations. Future BD research utilizing human cybrid lines, conplastic animals, and admixed human populations offer promising advancements in the field.

Experiments with human cybrids have shown that mtDNA representing populations from different geographic origins have differential OXPHOS efficiency and may play a role in susceptibilities to diseases (Lin et al., 2012; Kenney et al., 2014a, b). Incompatibilities between mtDNA and nuclear-encoded mitochondrial genes results in inefficient electron flow down the respiratory chain leading to increased release of free radicals, and a reduction of catalytic sites and ATP production (Lane, 2011; Meiklejohn et al., 2013). Mitochondrial haplogroups accumulate population-specific mtDNA variants which represent ancestral origins. Human cybrids studies using identical nuclei and common BD haplogroups can be used to investigate and potentially identify deleterious effects of mtDNA variants underlying bioenergetic deficiencies, altered OXPHOS metabolism, and deficient Ca^2+^ homeostasis when they are introduced into a new nuclear ancestral background lacking population specific nDNA suppressors.

Conplastic animal models can also help elucidate how mtDNA variation influences organismal pathophysiology and mitochondrial functioning. Previous studies investigating physiological and phenotypic variability influenced by mitochondria have reported that different mtDNA haplotypes can influence mitochondrial functioning, cellular processes, and behaviors that have been previously implicated in BD. These include the regulation of OXPHOS enzyme levels, reduced cellular ATP levels, pathological morphology of mitochondria, ROS generation, impaired glucose tolerance, reduced glucose-induced insulin secretion, and increased anxiety-related behavior, resulting in significant differences in health and longevity between various conplastic strains (Yu et al., 2009; Weiss et al., 2012; Houstek et al., 2014; Latorre-Pellicer et al., 2016).

Admixed populations, such as Hispanic and African Americans, also provide a unique opportunity to examine the role of mitonuclear discordance in BD, as they are genetically heterogeneous with varying proportions of European, African, and Native American ancestries (Bertoni et al., 2003). Therefore, mitonuclear incompatibility in these populations represents a possible disruption of 20,000–150,000 years of co-evolution between mitonuclear genomes. The increased genetic diversity in human admixed populations due to divergent underlying ancestral populations of mtDNA and nDNA may lead to increased collection and sharing of GWAS, transcriptome, and mtDNA sequence data from under-represented minority populations.

Research methods designed to capitalize on mitonuclear interactions and detect incompatibilities as outlined in this review can be applied to other psychiatric disorders, as mitochondrial dysfunction is well documented in schizophrenia (Goncalves et al., 2015; Hjelm et al., 2015; Monpays et al., 2016; Ben-Shachar, 2017; Ni and Chung, 2020; Roberts, 2020), depression (Bansal and Kuhad, 2016; Allen et al., 2018; Caruso et al., 2019; Forester et al., 2019), autism (Rossignol and Frye, 2012; Goh et al., 2014; Varga et al., 2018; Bennuri et al., 2019; Citrigno et al., 2020; Frye, 2020; Gevezova et al., 2020), and anxiety (Chakravarty et al., 2013; Khalifeh et al., 2016; Babenko et al., 2018). The studies outlined in this review suggest that mitochondrial dysfunction underlying BD and other psychiatric disorders with genetic overlap may be the consequence of mitonuclear incompatibility, and may be enhanced in admixed populations with mitonuclear genomes originating from different ancestral populations (Chou and Leu, 2015).

## Author Contributions

The author confirms being the sole contributor of this work and has approved it for publication.

## Conflict of Interest

The author declares that the research was conducted in the absence of any commercial or financial relationships that could be construed as a potential conflict of interest.
